# Prenatal valproic acid-induced autism marmoset model exhibits higher salivary cortisol levels

**DOI:** 10.3389/fnbeh.2022.943759

**Published:** 2022-08-11

**Authors:** Madoka Nakamura, Akiko Nakagami, Keiko Nakagaki, Miyuki Yasue, Nobuyuki Kawai, Noritaka Ichinohe

**Affiliations:** ^1^Department of Cognitive and Psychological Sciences, Graduate School of Informatics, Nagoya University, Nagoya, Japan; ^2^Laboratory for Ultrastructure Research, National Institute of Neuroscience, National Center of Neurology and Psychiatry, Kodaira, Japan; ^3^Department of Psychology, Japan Women’s University, Tokyo, Japan; ^4^Academy of Emerging Science, Chubu University, Kasugai, Japan

**Keywords:** VPA marmoset, cortisol, diurnal change, stress, autism spectrum disorder

## Abstract

Individuals with autism spectrum disorder (ASD) are exposed to a variety of stressors owing to their behavioral traits. Cortisol is a hormone typically associated with stress, and its concentration and response to stress are higher in individuals with ASD than in controls. The mechanisms underlying cortisol dysregulation in ASD have been explored in rodents. Although rodent models have successfully replicated the major symptoms of autism (i.e., impaired vocal communication, social interaction deficits, and restricted/repetitive patterns of behavior), evidence suggests that the hypothalamic-pituitary-adrenal (HPA) axis system differs between rodents and primates. We developed an ASD model in the common marmoset (*Callithrix jacchus*), a New World monkey, utilizing prenatal exposure to valproic acid (VPA). In this study, we collected the salivary cortisol levels in VPA-exposed and unexposed marmosets in the morning and afternoon. Our results revealed that both VPA-exposed and unexposed marmosets showed similar diurnal changes in cortisol levels, which were lower in the afternoon than in the morning. However, heightened cortisol levels were observed throughout the day in VPA-exposed marmosets. These results are consistent with those of ASD in humans. Our results suggest that VPA-exposed marmosets show similarities not only in their behavioral patterns and brain pathologies, which we have reported previously, but also in hormonal regulation, validating the usefulness of VPA-exposed marmosets also as a tool for ASD stress research.

## Introduction

Autism spectrum disorder (ASD) is a neurodevelopmental disorder with a prevalence of 1 in 44 (Maenner et al., 2021). The major symptoms of ASD are impaired vocal communication, deficits in social interaction, and restricted/repetitive behavior patterns (American Psychological Association [APA], and American Psychiatric Association [APA], 2013). Individuals with ASD are exposed to a variety of stressors owing to their behavioral traits, and overwhelming stress in ASD can trigger secondary deficits, such as depression and sleep disorders. Cortisol is a hormone typically associated with stress, and its concentration and response to stress are higher in individuals with ASD than in controls. The mechanisms underlying cortisol dysregulation in ASD have been explored in rodent models; however, differences in the regulatory system of the hypothalamic-pituitary-adrenal (HPA) axis in rodents and primates have been reported (Gibbs, 1986; Broadbear et al., 2004; Goursaud et al., 2006; Bertani et al., 2010). For example, rats are nocturnal and marmosets are diurnal like humans, and the circadian rhythms of serum hormone levels in rats and marmosets are different (Bertani et al., 2010). Cellular and molecular properties in hypothalamus are largely conserved but still show some differences in gene expression patterns (van Eerdenburg and Rakic, 1994; Zhou et al., 2020). Furthermore, in mice, cortisol suppresses heat production in adipose tissue, but the same is unlikely to occur in primates (Luijten et al., 2019). Considering translational research, the primate ASD model appears to be preferable for studying the hormonal stress response in ASD.

We have previously developed an ASD model for a New World monkey, the common marmoset (*Callithrix jacchus*) (Yasue et al., 2015, 2018; Watanabe et al., 2021). The marmosets were prenatally exposed to valproic acid (VPA), which is often used in rodent models of ASD. VPA epigenetically alters gene expression in the developing fetal brain. VPA-exposed marmosets demonstrated all three core symptoms of ASD: (1) biased usage of vocal repertoires, (2) weak social attention to unfamiliar conspecifics, and (3) deficits in reversal learning (Watanabe et al., 2021; Nakagami et al., 2022). In addition, when unfair rewards were given for the same task between two marmosets (Yasue et al., 2018), VPA-exposed marmosets continued the task even when it was unfavorable to them, suggesting a lack of attention to the coproducing species (weak inequity aversion). They also failed to recognize differences between third-party reciprocal and non-reciprocal exchanges, whereas VPA-unexposed marmosets (UE marmosets) discriminated against these exchanges (Kawai et al., 2014, 2019; Yasue et al., 2015). Furthermore, transcriptome analyses have revealed that VPA-exposed marmosets replicate a broad range of gene dysregulation in human idiopathic ASD, whereas rodent models generally replicate only a smaller part of the pathology (Watanabe et al., 2021). Especially, VPA-exposed marmosets recapitulate human ASD well in the four major cell types of the brain, consisting of neurons and three types of glia, but less well in glia in rodents. Thus, VPA-exposed marmosets appear to provide a suitable model for translational research on ASD.

In this study, we collected the salivary cortisol from marmosets in the morning and afternoon. Salivary measurements of cortisol have been shown to closely mirror those in the serum (Perogamvros et al., 2010; VanBruggen et al., 2011). We found that VPA-exposed marmosets had heightened basal cortisol levels, as do humans with ASD. Our results suggest that VPA-exposed marmosets may be useful for translational research on stress pathophysiology in ASD.

## Materials and methods

### Subjects

All experimental animal care procedures were conducted under approved protocols according to the regulations of the National Center of Neurology and Psychiatry (NCNP), Tokyo, Japan. Ten UE marmosets (five males and five females) and nine VPA-exposed marmosets (five males and four females) were included in this study (Table 1). The ages of the experimental animals range from 2- to 8-year-old. The mean age was 4.7 ± 1.62 years (UE marmosets: 4.5 ± 1.43, VPA-exposed marmosets: 4.9 ± 1.79). The subjects were born and raised in family cages. Then, at least 3 months prior to the experiment, they were transferred to individual stainless steel home cages (Natsume Manufacturing Co., Ltd., Tokyo, Japan) in order to avoid the confounding effect on cortisol levels due to interactions with conspecifics of the same cage, which are difficult to control. The subjects were kept at room temperature of 29 ± 2°C and maintained on a 12 h:12 h light:dark cycle with free access to food and water. The lights in the breeding room were turned on at 7:00 a.m. and turned off at 7:00 p.m. daily. Marmosets in the facility were familiar with human contact and approached experimenters to obtain food rewards without hesitation.

**TABLE 1:** Subject information.

Animal ID	Siblings	Age (year)	Sex	Group
11111		8	Female	UE
12043	$	7	Female	VPA
12044	$	7	Female	VPA
12062		7	Female	VPA
13019		6	Female	UE
14002		5	Female	VPA
14030	#	5	Male	VPA
14031	#	5	Male	VPA
14078		5	Male	UE
15033	%	4	Male	UE
15034	%	4	Male	UE
15041		4	Female	UE
15049	+	4	Female	UE
15050	+	4	Male	UE
16043		3	Male	VPA
16086		3	Female	UE
16089		3	Male	UE
16152		3	Male	VPA
17024		2	Male	VPA

2- to 8-year-old (n = 10 males, n = 9 females) adult marmosets were used in this study. The mean age of subjects (n = 19) in the study was 4.7 ± 1.62 years. Animals with the same symbol in the “siblings” column are littermates.

### Valproic acid treatment

Valproic acid marmosets were exposed to valproic acid during their fetal stage, whereas UE marmosets were not (Yasue et al., 2015). The dams of VPA-exposed marmosets were housed in their cages. Their blood progesterone levels were monitored periodically to determine the timing of pregnancy, as was done for the UE dams. The VPA group received 200 mg/kg intragastric sodium valproate via an oral catheter daily on days 60 to 66 after conception, for a total of seven treatments. This period was determined based on the administration period (E12 of the rat fetus) used to produce VPA-exposed rodent models of ASD. All VPA dams received the medication without vomiting and showed no signs of abnormal pregnancy or delivery. The dams of UE marmosets were administered neither VPA nor a solvent during this period to prevent miscarriage. VPA marmosets displayed no malformations or body weight differences compared with UE marmosets.

### Salivary collection and assay

Saliva was sampled to measure cortisol levels twice daily at 7:30 a.m. and at 6:30 p.m., as described in previous research (Kaplan et al., 2012). Animals were not captured by the experimenter and saliva was collected under free-ranging conditions in their home cages. A thin cotton swab (Matsumotokiyoshi Co., Ltd., Chiba, Japan) was used to collect saliva from the marmosets. The marmosets were fully trained to bite cotton swabs in their home cages before the experiments. The cotton swabs were dipped in powdered sugar to ensure that the bites would last sufficiently long (approximately 3–5 min) (Kaplan et al., 2012). A 2.0-mL Costar Spin-X centrifuge tube with a nylon filter (0.22 μm) was filled with the swabs and centrifuged at 10,000 rpm for 5 min to extract the liquid portion of the sample. The collected samples were stored in a −80°C freezer until further use. Saliva samples were collected three times per individual, with no two collections occurring in the same month. The duration of the experiment was 3 months, from June to August in 2019. Cortisol levels (μg/dl) were measured using an AIA-360 Automated Immunoassay Analyzer with AIA-pack cortisol test cups (Tosoh Corporation, Tokyo, Japan) and averaged per subject across all saliva collections either in the morning or the evening.

### Statistical analysis

Two-factor repeated-measures ANOVA and student-*t* test were performed using JMP 16 software (SAS Institute, Cary, NC, United States) when the data were determined to be normally distributed by the Shapiro–Wilk test or by the *F*-test. If we found that the data showed a non-normal distribution, a mixed design ANOVA by a downloadable program (ARTool) (Wobbrock et al., 2018) was performed using R. *P*-values less than 0.05 were considered statistically significant.

## Results

Salivary samples were successfully collected by experimenters while the marmosets were in their home cages. They showed no signs of aggression or aversion during sampling. No significant effect of sex on salivary cortisol levels was observed in both UE and VPA-exposed groups in the morning and evening. Therefore, we analyzed the data for male and female together.

**FIGURE 1:**
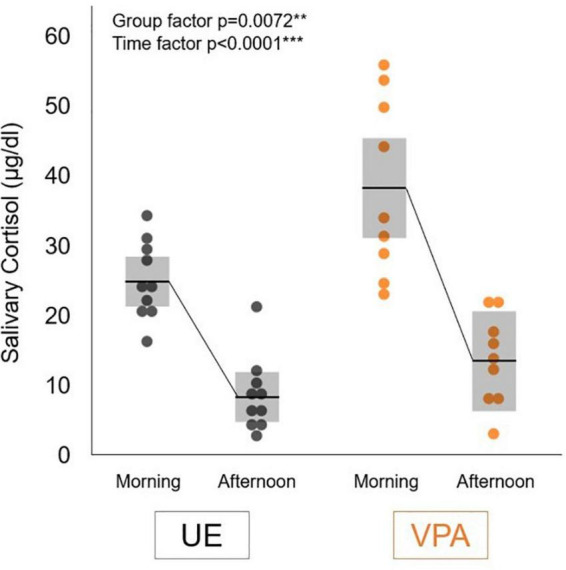
Salivary cortisol levels in the morning (7:30 a.m.) and afternoon (6:30 p.m.) in the valproic acid-exposed (VPA group) and unexposed (UE group) marmosets. Values are expressed as mean ± standard deviation (*n* = 10 for UE group, *n* = 9 for VPA group, **p < 0.01, ***p < 0.001).

Figure 1 shows group salivary cortisol levels (mean ± SD) measured in the morning (7:30 a.m.) and afternoon (6:30 p.m.). Two-factor repeated measures ANOVA showed a significant difference in the group factor [*F*(1,17) = 9.3153, *p* = 0.0072] and time factor [*F*(1,17) = 100.8143, *p* < 0.0001]. We did not find any significant difference in interaction effects [*F*(1,17) = 4.0257, *p* = 0.0610]. These results suggested that the VPA-exposed marmosets maintained diurnal changes with high cortisol levels in the morning that fell throughout the afternoon, and that salivary cortisol levels in the VPA-exposed group were significantly higher than those in the UE group at the times examined in this study.

We have also examined the correlation between age and salivary cortisol level. Although no statistically significant correlation between age and cortisol level was found at any time of day or in any group, there was a positive correlation in UE marmosets in the afternoon (*r* = 0.58) and a weak positive correlation in VPA-exposed marmosets (*r* = 0.32). In the morning, no correlation between age and salivary cortisol level was found for both groups.

Unfortunately, we found that among the VPA-exposed marmosets used in the current experiment, only three VPA-exposed marmosets were involved in the task presented in the previous papers (Yasue et al., 2015, 2018; Nakagami et al., 2022). Among these three animals, one marmoset showed the lowest morning salivary cortisol level (23.1 μg/dL). This level was half that of the other two VPA-exposed marmosets and was similar to the average level of the UE marmoset. This VPA-exposed marmoset had the best performance in three social tasks (social gazing, inequity aversion, and third-party reciprocity) among these three VPA-exposed marmosets. These anecdotal observations suggest that there may be a relationship between cortisol levels and levels of social impairment in VPA-exposed marmosets.

Of the animals in this experiment, there were two pairs of siblings in UE marmosets and two pairs in VPA marmosets (Table 1). None of these pairs had cortisol levels biased in one direction relative to the mean in both morning and evening (data not shown), suggesting that the results in this study were not biased due to the inclusion of siblings.

Note that saliva sampling in this study was from marmosets that were in individual cages, so the results of this study may include isolation-related stress. We considered the possibility that the time between the animals’ transfer to the single cage and the sampling start point could affect the results of this study. First, there was no significant difference in the time from isolation to the experiment between UE and VPA-exposed marmosets (*p* = 0.8659, student *t*-test). We also examined temporal changes of cortisol level in samples at three time points taken from each animal separately in the morning and afternoon. A mixed design ANOVA showed a significant difference only in the group factor [*F*(1,16) = 7.6731, *p* = 0.0131] in the morning cortisol level, but not in the time factor and interaction effects in the morning or afternoon. The results indicate that there is no specific pattern of variation in cortisol level among three samples over 3 months in either UE or VPA, suggesting the effect of isolation time before the experiment is minimal in both groups.

## Discussion

This study revealed that both UE- and VPA-exposed marmosets showed a similar diurnal change in cortisol levels, which was lower in the afternoon than in the morning. This is consistent with the circadian rhythm of cortisol levels in humans (Smyth et al., 1997). However, heightened cortisol levels were observed in VPA-exposed marmosets throughout the day. Previous studies have shown that VPA-exposed marmosets use phee calls (isolation call) more frequently than do UE marmosets (Yamaguchi et al., 2010; Watanabe et al., 2021). This suggests that VPA-exposed marmosets may be in a constant state of stress, such as anxiety.

Studies of cortisol levels in humans with ASD have shown inconsistent results (Tordjman et al., 2014). The discrepancies in their results may be related to study methods (plasma cortisol measures vs. urinary or salivary measures), and sample sizes. We thus compared our results with those of five studies that used a sufficient number of cases with salivary sampling in humans (more than 20 cases in both control and ASD groups). Four of those reports showed elevated cortisol concentrations (Corbett et al., 2008; Kidd et al., 2012; Spratt et al., 2012; Tordjman et al., 2014). One report commented on the high variability of cortisol concentration in ASD cases (Corbett et al., 2009). In studies examining diurnal variation, two reports observed cortisol increases in the morning and evening (Corbett et al., 2008; Tordjman et al., 2014), while another observed cortisol increases only in the morning (Kidd et al., 2012). Thus, our results are consistent with these reports. VPA-exposed marmosets replicated the abnormal endocrine function observed in people with ASD.

Among rodent models of ASD, BTBR mice and VPA-exposed male rats showed heightened serum cortisol levels (Schneider et al., 2008; Benno et al., 2009; Frye and Llaneza, 2010; Silverman et al., 2010; Ferraro et al., 2021), similar to VPA-exposed marmosets and people with idiopathic ASD. In contrast, transgenic ASD model mice (MeCP2 and FMR1 mutant) showed no elevated levels of cortisol (McGill et al., 2006; Qin and Smith, 2008). It can be difficult, however, to collect specimens for cortisol measurement in rodents without stressing the animals. To avoid this problem, blood is often collected from the heart immediately after euthanasia via acute decapitation. Collection of salivary cortisol or plasma cortisol using the tail cutting technique also requires restraint and is unsuitable for measuring basal cortisol levels (Fenske, 1997; Kim et al., 2018). Blood sampling through an intravenous catheter in large vessels can be applicable for plasma cortisol measurement on freely moving rodents (Nyuyki et al., 2012). However, this technique is still invasive and not suitable for long-term cortisol level monitoring. In this study, we established a method to collect saliva from marmosets after acclimatization by training. The current procedure using marmosets will allow the repeated examination of cortisol levels in ASD models in the same individuals, both at basal levels and in the stress response, and will contribute to a reduction in the number of experimental animals.

This study revealed that VPA-exposed marmosets reproduced the variability of cortisol levels in human ASD. Marmosets are cooperative and highly social primates and are considered a suitable model animal to study stress in social life, and they also show higher social function deficits as in cases of ASD (Yasue et al., 2015, 2018; Watanabe et al., 2021; Nakagami et al., 2022). Further examination of cortisol levels in VPA-exposed marmosets would provide a new avenue for studying the biology of stress faced by individuals with ASD and for developing novel therapeutic interventions.

## Data availability statement

The original contributions presented in this study are included in the article/supplementary material. Further inquiries can be directed to the corresponding authors.

## Ethics statement

This animal study was reviewed and approved by the regulations of the National Center of Neurology and Psychiatry (NCNP), Tokyo, Japan.

## Author contributions

NK, NI, MN, and AN designed this study. MN, AN, and MY performed salivary sampling. MN analyzed the data. KN managed the production and physical condition of the animals. NK, NI, and MN wrote the manuscript. All authors have read and approved the final manuscript.

## References

[B1] American Psychological Association [APA], and American Psychiatric Association [APA] (2013). *Diagnostic and Statistical Manual of Mental Disorders: DSM-5.* Arlington, TX: American Psychological Association.

[B2] Benno R. Smirnova Y. Vera S. Liggett A. Schanz N. (2009). Exaggerated responses to stress in the BTBR T+tf/J mouse: an unusual behavioral phenotype. *Behav. Brain Res.* 197 462–465. 10.1016/j.bbr.2008.09.041 18977396

[B3] Bertani S. Carboni L. Criado A. Michielin F. Mangiarini L. Vicentini E. (2010). Circadian profile of peripheral hormone levels in Sprague-Dawley rats and in common marmosets (*Callithrix jacchus*). *In Vivo* 24 827–836. 21164040

[B4] Broadbear J. H. Winger G. Woods J. H. (2004). Self-administration of fentanyl, cocaine and ketamine: effects on the pituitary-adrenal axis in rhesus monkeys. *Psychopharmacology (Berl)* 176 398–406. 10.1007/s00213-004-1891-x 15114434

[B5] Corbett B. A. Mendoza S. Wegelin J. A. Carmean V. Levine S. (2008). Variable cortisol circadian rhythms in children with autism and anticipatory stress. *J. Psychiatry Neurosci.* 33 227–234. 18592041 PMC2441887

[B6] Corbett B. A. Schupp C. W. Levine S. Mendoza S. (2009). Comparing cortisol, stress, and sensory sensitivity in children with autism. *Autism Res.* 2 39–49. 19358306 10.1002/aur.64 PMC2698454

[B7] Fenske M. (1997). The use of salivary cortisol measurements for the non-invasive assessment of adrenal cortical function in Guinea pigs. *Exp. Clin. Endocrinol. Diabetes* 105 163–168. 10.1055/s-0029-1211746 9228513

[B8] Ferraro S. De Zavalia N. Belforte N. Amir S. (2021). In utero exposure to valproic-acid alters circadian organisation and clock-gene expression: implications for autism spectrum disorders. *Front. Behav. Neurosci.* 15:711549. 10.3389/fnbeh.2021.711549 34650409 PMC8505722

[B9] Frye C. A. Llaneza D. C. (2010). Corticosteroid and neurosteroid dysregulation in an animal model of autism, BTBR mice. *Physiol. Behav.* 100 264–267. 10.1016/j.physbeh.2010.03.005 20298706 PMC2860004

[B10] Gibbs D. M. (1986). Vasopressin and oxytocin: hypothalamic modulators of the stress response: a review. *Psychoneuroendocrinology* 11 131–139. 10.1016/0306-4530(86)90048-x 3018820

[B11] Goursaud A.-P. S. Mendoza S. P. Capitanio J. P. (2006). Do neonatal bilateral ibotenic acid lesions of the hippocampal formation or of the amygdala impair HPA axis responsiveness and regulation in infant rhesus macaques (*Macaca mulatta*)? *Brain Res.* 1071 97–104. 10.1016/j.brainres.2005.11.027 16412391

[B12] Kaplan G. Pines M. K. Rogers L. J. (2012). Stress and stress reduction in common marmosets. *Appl. Anim. Behav. Sci.* 137 175–182.

[B13] Kawai N. Nakagami A. Yasue M. Koda H. Ichinohe N. (2019). Common marmosets (*Callithrix jacchus*) evaluate third-party social interactions of human actors but Japanese monkeys (*Macaca fuscata*) do not. *J. Comparat. Psychol.* 133:488. 10.1037/com0000182 31021114

[B14] Kawai N. Yasue M. Banno T. Ichinohe N. (2014). Marmoset monkeys evaluate third-party reciprocity. *Biol. Lett.* 10:20140058. 10.1098/rsbl.2014.0058 PMC4046368 24850892

[B15] Kidd S. A. Corbett B. A. Granger D. A. Boyce W. T. Anders T. F. Tager I. B. (2012). Daytime secretion of salivary cortisol and alpha-amylase in preschool-aged children with autism and typically developing children. *J. Autism Dev. Disord.* 42 2648–2658. 10.1007/s10803-012-1522-z 22477468 PMC3602445

[B16] Kim S. Foong D. Cooper M. S. Seibel M. J. Zhou H. (2018). Comparison of blood sampling methods for plasma corticosterone measurements in mice associated with minimal stress-related artefacts. *Steroids* 135 69–72. 10.1016/j.steroids.2018.03.004 29548771

[B17] Luijten I. H. Cannon B. Nedergaard J. (2019). Glucocorticoids and brown adipose tissue: do glucocorticoids really inhibit thermogenesis? *Mol. Aspects Med.* 68 42–59. 10.1016/j.mam.2019.07.002 31323252

[B18] Maenner M. J. Shaw K. A. Bakian A. V. Bilder D. A. Durkin M. S. Esler A. (2021). Prevalence and characteristics of autism spectrum disorder among children aged 8 years—autism and developmental disabilities monitoring network, 11 sites, United States, 2018. *MMWR Surveill. Summ.* 70:1. 10.15585/mmwr.mm6745a7 34855725 PMC8639024

[B19] McGill B. E. Bundle S. F. Yaylaoglu M. B. Carson J. P. Thaller C. Zoghbi H. Y. (2006). Enhanced anxiety and stress-induced corticosterone release are associated with increased Crh expression in a mouse model of Rett syndrome. *Proc. Natl. Acad. Sci. U. S. A.* 103 18267–18272. 10.1073/pnas.0608702103 17108082 PMC1636379

[B20] Nakagami A. Yasue M. Nakagaki K. Nakamura M. Kawai N. Ichinohe N. (2022). Reduced childhood social attention in an autism model marmoset predicts impaired social skills and inflexible behavior in adulthood. *Front. Psychiatry* 13:885433. 10.3389/fpsyt.2022.885433 35958665 PMC9357878

[B21] Nyuyki K. D. Maloumby R. Reber S. O. Neumann I. D. (2012). Comparison of corticosterone responses to acute stressors: chronic jugular vein versus trunk blood samples in mice. *Stress* 15 618–626. 10.3109/10253890.2012.655348 22251167

[B22] Perogamvros I. Keevil B. G. Ray D. W. Trainer P. J. (2010). Salivary cortisone is a potential biomarker for serum free cortisol. *J. Clin. Endocrinol. Metab.* 95 4951–4958. 10.1210/jc.2010-1215 20685855

[B23] Qin M. Smith C. B. (2008). Unaltered hormonal response to stress in a mouse model of fragile X syndrome. *Psychoneuroendocrinology* 33 883–889. 10.1016/j.psyneuen.2008.03.010 18479837 PMC2615669

[B24] Schneider T. Roman A. Basta-Kaim A. Kubera M. Budziszewska B. Schneider K. (2008). Gender-specific behavioral and immunological alterations in an animal model of autism induced by prenatal exposure to valproic acid. *Psychoneuroendocrinology* 33 728–740. 10.1016/j.psyneuen.2008.02.011 18396377

[B25] Silverman J. L. Yang M. Turner S. M. Katz A. M. Bell D. B. Koenig J. I. (2010). Low stress reactivity and neuroendocrine factors in the BTBR T+tf/J mouse model of autism. *Neuroscience* 171 1197–1208. 10.1016/j.neuroscience.2010.09.059 20888890 PMC2991427

[B26] Smyth J. M. Ockenfels M. C. Gorin A. A. Catley D. Porter L. S. Kirschbaum C. (1997). Individual differences in the diurnal cycle of cortisol. *Psychoneuroendocrinology* 22 89–105. 9149331 10.1016/s0306-4530(96)00039-x

[B27] Spratt E. G. Nicholas J. S. Brady K. T. Carpenter L. A. Hatcher C. R. Meekins K. A. (2012). Enhanced cortisol response to stress in children in autism. *J. Autism Dev. Disord.* 42 75–81. 10.1007/s10803-011-1214-0 21424864 PMC3245359

[B28] Tordjman S. Anderson G. M. Kermarrec S. Bonnot O. Geoffray M.-M. Brailly-Tabard S. (2014). Altered circadian patterns of salivary cortisol in low-functioning children and adolescents with autism. *Psychoneuroendocrinology* 50 227–245. 10.1016/j.psyneuen.2014.08.010 25244637

[B29] van Eerdenburg F. J. Rakic P. (1994). Early neurogenesis in the anterior hypothalamus of the rhesus monkey. *Dev. Brain Res.* 79 290–296. 10.1016/0165-3806(94)90134-1 7955328

[B30] VanBruggen M. D. Hackney A. C. McMurray R. G. Ondrak K. S. (2011). The relationship between serum and salivary cortisol levels in response to different intensities of exercise. *Int. J. Sports Physiol. Perform.* 6 396–407. 10.1123/ijspp.6.3.396 21911864

[B31] Watanabe S. Kurotani T. Oga T. Noguchi J. Isoda R. Nakagami A. (2021). Functional and molecular characterization of a non-human primate model of autism spectrum disorder shows similarity with the human disease. *Nat. Commun.* 12:5388. 10.1038/s41467-021-25487-6 34526497 PMC8443557

[B32] Wobbrock J. O. Findlater L. Gergle D. Higgins J. J. Kay M. (2018). *ARTool Align-and-Rank Data for A Nonparametric ANOVA.* Available online at: https://deptswashingtonedu/aimgroup/proj/art/ (accessed July 1, 2022)

[B33] Yamaguchi C. Izumi A. Nakamura K. (2010). Time course of vocal modulation during isolation in common marmosets (*Callithrix jacchus*). *Am. J. Primatol.* 72 681–688. 10.1002/ajp.20824 20301139

[B34] Yasue M. Nakagami A. Banno T. Nakagaki K. Ichinohe N. Kawai N. (2015). Indifference of marmosets with prenatal valproate exposure to third-party non-reciprocal interactions with otherwise avoided non-reciprocal individuals. *Behav. Brain Res.* 292 323–326. 10.1016/j.bbr.2015.06.006 26133500

[B35] Yasue M. Nakagami A. Nakagaki K. Ichinohe N. Kawai N. (2018). Inequity aversion is observed in common marmosets but not in marmoset models of autism induced by prenatal exposure to valproic acid. *Behav. Brain Res.* 343 36–40. 10.1016/j.bbr.2018.01.013 29374522

[B36] Zhou X. Zhong S. Peng H. Liu J. Ding W. Sun L. (2020). Cellular and molecular properties of neural progenitors in the developing mammalian hypothalamus. *Nat. Commun.* 11 1–16. 32792525 10.1038/s41467-020-17890-2 PMC7426815

